# Optimizing older patient care in emergency departments: a comprehensive survey of current practices and challenges in Northern Italy

**DOI:** 10.1186/s12873-024-01004-y

**Published:** 2024-05-20

**Authors:** Elena Pinardi, Alice Margherita Ornago, Angelo Bianchetti, Alessandro Morandi, Stefano Mantovani, Alessandra Marengoni, Mauro Colombo, Beatrice Arosio, Chukwuma Okoye, Francesca Cortellaro, Giuseppe Bellelli

**Affiliations:** 1 School of Medicine and Surgery, University of Milano-Bicocca, Milan, Italy; 2 Italian Society of Gerontology and Geriatrics (Società Italiana di Gerontologia e Geriatria - SIGG), Firenze, Italy; 3 Medicine and Rehabilitation Department, Istituto Clinico S.Anna Hospital, Gruppo San Donato, Brescia, Italy; 4 Intermediate Care and Rehabilitation, Azienda Speciale “Cremona Solidale”, Cremona, Italy; 5 Parc Sanitari Pere Virgili, Vall d’Hebrón Institute of Research, Barcelona, Spain; 6 RSA Don Giuseppe Cuni, Magenta, Italy; 7 Department of Clinical and Experimental Sciences, Geriatric Unit, University of Brescia, Brescia, Italy; 8 Golgi Cenci Foundation, Abbiategrasso, Italy; 9 Department of Clinical Sciences and Community Health, University of Milan, Milan, Italy; 10 Integrazione Percorsi di Cura Ospedale Territorio, Urgency Emergency Regional Agency (Agenzia Regionale Emergenza Urgenza - AREU), Milan, Italy; 11 Acute Geriatrics Unit, Fondazione IRCCS San Gerardo dei Tintori, Monza, Italy

**Keywords:** Survey, Older adults, Emergency department, Geriatric care

## Abstract

**Background:**

The progressive aging of the population and the increasing complexity of health issues contribute to a growing number of older individuals seeking emergency care. This study aims to assess the state of the art of care provided to older people in the Emergency Departments of Lombardy, the most populous region in Italy, counting over 2 million people aged 65 years and older.

**Methods:**

An online cross-sectional survey was developed and disseminated among emergency medicine physicians and physicians affiliated to the Lombardy section of the Italian Society of Geriatrics and Gerontology (SIGG), during June and July 2023. The questionnaire covered hospital profiles, geriatric consultation practices, risk assessment tools, discharge processes and perspectives on geriatric emergency care.

**Results:**

In this mixed method research, 219 structured interviews were collected. The majority of physicians were employed in hospitals, with 54.7% being geriatricians. Critical gaps in older patient’s care were identified, including the absence of dedicated care pathways, insufficient awareness of screening tools, and a need for enhanced professional training.

**Conclusions:**

Tailored protocols and geriatric educational programs are crucial for improving the quality of emergency care provided to older individuals. These measures might also help relieve the burden on the Emergency Departments, thereby potentially enhancing overall efficiency and ensuring better outcomes.

**Supplementary Information:**

The online version contains supplementary material available at 10.1186/s12873-024-01004-y.

## Background

Demographic changes have led to increasing numbers of older adults seeking emergency care worldwide [1]. Older adults attending the Emergency Department (ED) are often characterized by multiple comorbidities, polypharmacy, impaired cognitive capacities, functional impairment, and multi-domain problems [2–4]. Moreover, disease presentation is frequently atypical compared to adults, further challenging timely diagnosis and adequate treatment [5, 6]. Co-occurrence of multiple diseases coupled with a high prevalence of geriatric syndromes and atypical complaints often make protocol-driven approaches unsuitable and result in an extended diagnostic workup [7, 8].

As a result, older individuals have a higher risk of experiencing prolonged stays, misdiagnoses, adverse health outcomes and unplanned revisits, placing a significant strain on both human and economic resources [2, 9]. Indeed, prior research reported a considerable burden for emergency physicians providing care to older adults, primarily attributed to factors such as inadequate training in geriatric medicine and limited experiences with this patient population [9].

Nonetheless, ED referral to older patients seems to be mostly appropriate, according to a previous national-based study carried out on more than 20 million referrals to the ED in Italy [10].

Despite the growing recognition of their specific needs in emergency care settings, there remains a noticeable gap in the existing literature regarding the comprehensive assessment and management of older individuals within the ED [11–15]. While international guidelines increasingly advocate for tailored care processes for this patient population, the adoption of these recommendations in real-world ED settings has not been comprehensively documented [16–18]. The limited adoption of interventions employing comprehensive geriatric assessments adapted for the ED suggests a need to further explore the potential barriers and facilitators to the implementation of these strategies.

With over 2 million people aged 65 and above, Lombardy hosts the highest number of older individuals in absolute terms among the Italian regions [19]. Its demographic profile, combined with its geographic characteristics, makes it comparable to an independent nation. In 2022, Lombardy’s ED services recorded nearly 3.5 million patient visits, exceeding the total for Belgium and Sweden in the same year [20, 21]. According to the regional portal EUOL (Emergency Urgency OnLine), approximately 28% of these visits were represented by individuals over 65 years, and this number is expected to further increase in the coming decades.

In this context, the study aims to provide a comprehensive view on the current state of care provided to older individuals presenting at the EDs in Lombardy, Italy. In addition, it aims to compare perspectives and practices of geriatricians, emergency medicine and other specialists, identifying key patterns, and delineating potential areas for improvement.

## Methods

### Study design

A cross-sectional survey was conducted among physicians affiliated to the Lombardy section of the Italian Society of Geriatrics and Gerontology (SIGG), as well as emergency medicine physicians within Lombardy. Participants were selected by convenience to capture different perspectives and offer a comprehensive understanding of the topic under investigation, enabling the creation of a diverse sample of key elements to the research question. Participants were reached through email invitations to participate voluntarily and anonymously.

### Questionnaire

For the purpose of this study, an exploratory approach was chosen. The survey content, informed by relevant literature in the broader field of geriatric emergency medicine [7, 22–26], was developed by a group of geriatricians and geriatric medicine residents at the University of Milano-Bicocca. Four major themes were addressed: patient-centered approach, unmet needs, continuity of care, competencies in geriatric emergency medicine.

The questionnaire was implemented using REDCap, an online management system for creating and publishing forms available to the public (https://projectredcap.org). Three of the authors considered expert in the field (GB, AB and AM) gave feedback on face and content validity of each single question and on the overall questionnaire, which was then modified in light of this feedback and distributed to all the study authors for pilot testing. Focus group discussions were used to evaluate readability, comprehensibility, duration, and completeness. The English translation of the final version of the survey is reported in Additional file 1.

The questionnaire consisted of 24 questions divided into 6 sections. Sections 1 and 2 focused on the physicians’ sample characteristics and hospital profiles and operational standards, respectively. Section 3 examined the presence of a consultant geriatrician, along with the frequency and reasons behind consultation requests in the ED. In sect. 4 screening tools for geriatric risk profiles were explored. Section 5 covered admission and discharge processes. Finally, sect. 6 assessed general perspectives on geriatric emergency care and the proficiency of geriatric-skilled staff. Sections 3, 5 and 6 employed five-point Likert scales for measurement. Open-ended questions were avoided except when necessary (age) to allow direct coding and standardized results.

### Data collection

Data were collected between June 6 and July 28, 2023.

### Statistical analysis

Only data from fully answered questionnaires were analyzed and reported. Quantitative data are presented as mean (standard deviation - SD) or median (interquartile range - IQR) as appropriate, and qualitative data as count (percentage). Stratified analyses based on participants’ specialty were performed using Chi-square test and one-way ANOVA to explore differences between the groups. Statistical significance was set at *P* < 0.05 in two-tailed tests.

All analyses were performed in R software, version 4.1.1 [27, 28].

This manuscript is reported using the Consensus-Based Checklist for Reporting of Survey Studies (CROSS) [29].

## Results

Two hundred and seventy-eight individuals participated in the survey. Among these, 50 were excluded due to incomplete data, 5 because they reported working outside Lombardy, and an additional 4 were excluded as they were not physicians. Therefore, the final sample included 219 questionnaires available for analysis. The demographic characteristics of the final sample are shown in Table 1.

**Table 1:** Main characteristics of the sample

Characteristic	N (%) or mean (SD)
Female sex	125 (57.1)
Age in years	44.7 (13.3)
Professional title	
Specialist physician	147 (67.1)
Resident physician	56 (25.6)
Physician without residency	10 (4.6)
Other	6 (2.7)
Specialty field*	
Geriatrics	111 (54.7)
Emergency Medicine	31 (15.3)
Internal Medicine	29 (14.3)
Other	48 (15.7)
Length of working experience	
< 5 years	69 (31.5)
5 to 9 years	22 (10.0)
10 to 20 years	46 (21.0)
> 20 years	82 (37.4)
Job setting	
Acute Hospital Ward	72 (32.9)
Emergency Department	68 (31.1)
Nursing Home	31 (14.2)
Rehabilitation Facility	14 (6.4)
Primary Care	14 (6.4)
Other	20 (9.1)
Work in an hospital	161 (73.5)
University-related/affiliated	90 (55.9)
With an Emergency Department	144 (89.4)

* *N* = 203

### Characteristics of survey participants

The mean age of survey participants was 44.7 (SD = 13.3) years, and 125 were women (57.1%). Most responders reported practicing in acute care wards and EDs (32.9 and 31.1%, respectively). Participants were predominantly geriatricians (54.7%), followed by emergency medicine specialists (15.3%) and internal medicine specialists (14.3%). Among those indicating employment in hospitals (73.5%), the majority reported to work in facilities equipped with EDs (55.9%).

### ED operational standards

Concerning operational standards within EDs, 66.7% of the participants reported a lack of specific care pathways for managing older patients; approximately 28% had either established or were in the process of planning such pathways, primarily outlined through formal written procedures. Furthermore, 92.7% of respondents supported the development of pathways specifically tailored for patients from nursing homes.

### Frequency and reasons for geriatric ED consulting

**Fig. 1:**
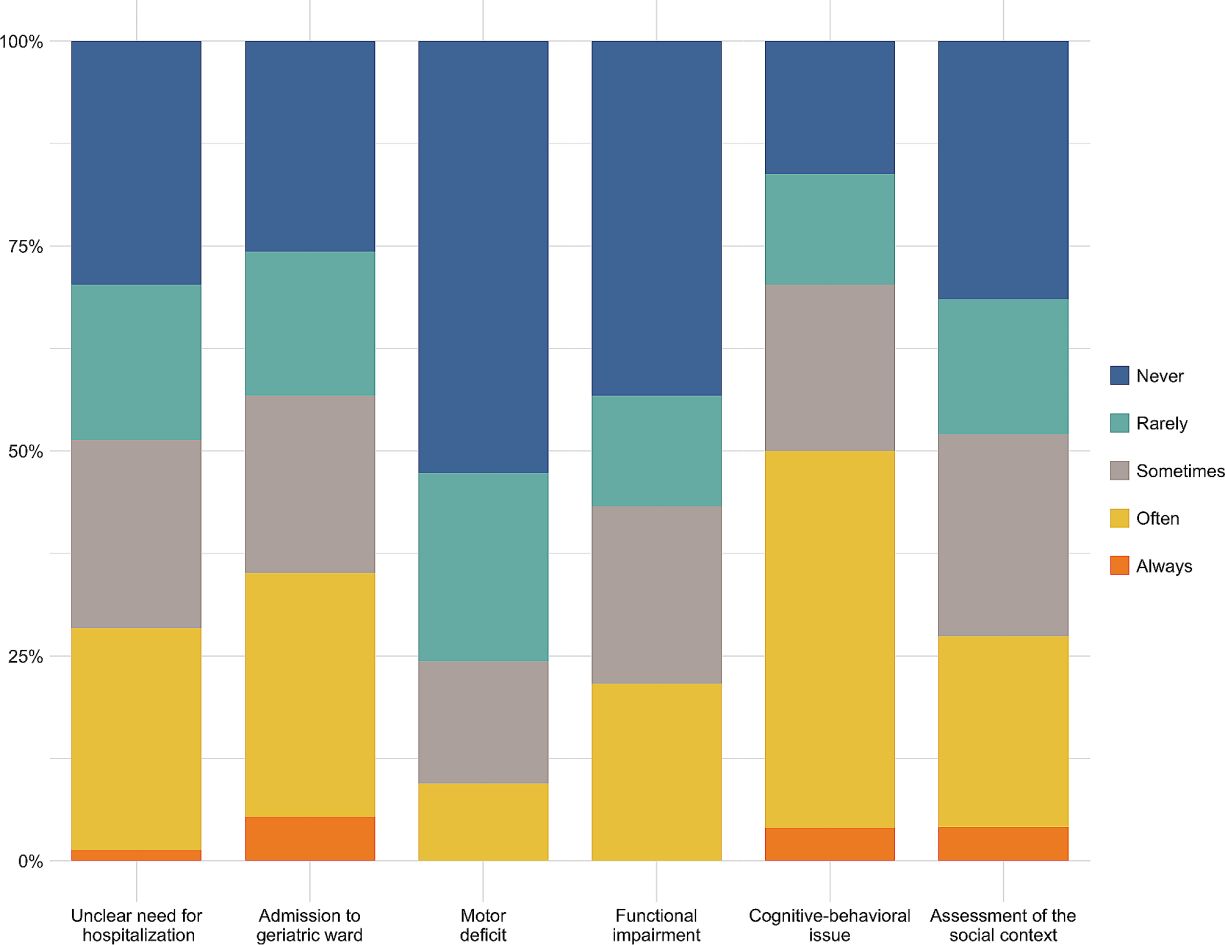
Stacked bar chart showing the proportion of responses regarding primary reasons for geriatric consultation in Emergency Departments (n = 72). Note: responses were provided on a 5-point Likert scale ranging from 1 *(never)* to 5 *(always)*

Approximately 46% of the physicians reported absence of a consultant geriatrician in their hospital or reference hospital. Among those with access to geriatric consulting, 36.6% had availability limited to daytime, while 6.2% had access only a few days a week; continuous presence was provided in just 4.3% of cases. The frequency of consultation requests also varied widely. After excluding responses from geriatricians directly working in the ED, approximately 26% of participants reported that consultant geriatricians were never called, 20.5% reported daily calls, and around 18% reported at least two calls per week. Cognitive-behavioral issues (such as dementia, agitation, or confusion) were the most common reasons for geriatric consulting, followed by requests for admission to geriatric wards. Conversely, motor deficits or functional impairment were infrequent causes (Fig. 1).

### Utilization of geriatric risk screening tools in the EDs

The Silver Code emerged as the best known geriatric risk screening tool among geriatricians, closely followed by Inter-RAI. In contrast, 52% of emergency medicine specialists reported having no knowledge of any screening tool, with Inter-RAI being the most recognized option. Only 17.4% of the participants indicated familiarity with more than one tool.

Regarding the actual utilization of geriatric risk screening tools, a notable majority of physicians (41.6%) reported that none were being employed in the ED of their hospital or reference hospital, while 36.1% expressed uncertainty. The Silver Code was the most commonly applied tool, acknowledged in 20.5% of the cases. Respondents also reported that, when a tool is utilized, it is filled out by the ER nurse in 71.4% of the cases, by a doctor in 16.3% of the cases, and through automated compilation in only 2% of cases.

### Hospitalization determinants

Physicians identified comorbidities, delirium, and inadequate social support as the primary factors influencing the decision for hospitalization of older patients, regardless of acute illness severity. Notably, in analyses stratified by specialty, a significant difference emerged for delirium (*p* = 0.001) and recent fall (*p* = 0.046), with these factors being consistently rated higher by geriatricians with respect to others. These data are reported in Additional File 2.

### ED discharge pathways

In the majority of cases no nurse appeared to be engaged in the discharge processes from the ED. When nurses were involved, those specialized in assisted discharge were much more frequently engaged than generalist nurses (22.4% vs. 4.1% of the cases). However, in 17.8% of cases, both professionals were reported to be involved simultaneously. Direct transfers of patients from the ED to a nursing home were generally considered infrequent, constituting a consolidated practice for only 17.4% of participants.

### Perspectives on geriatric care provision

**Fig. 2:**
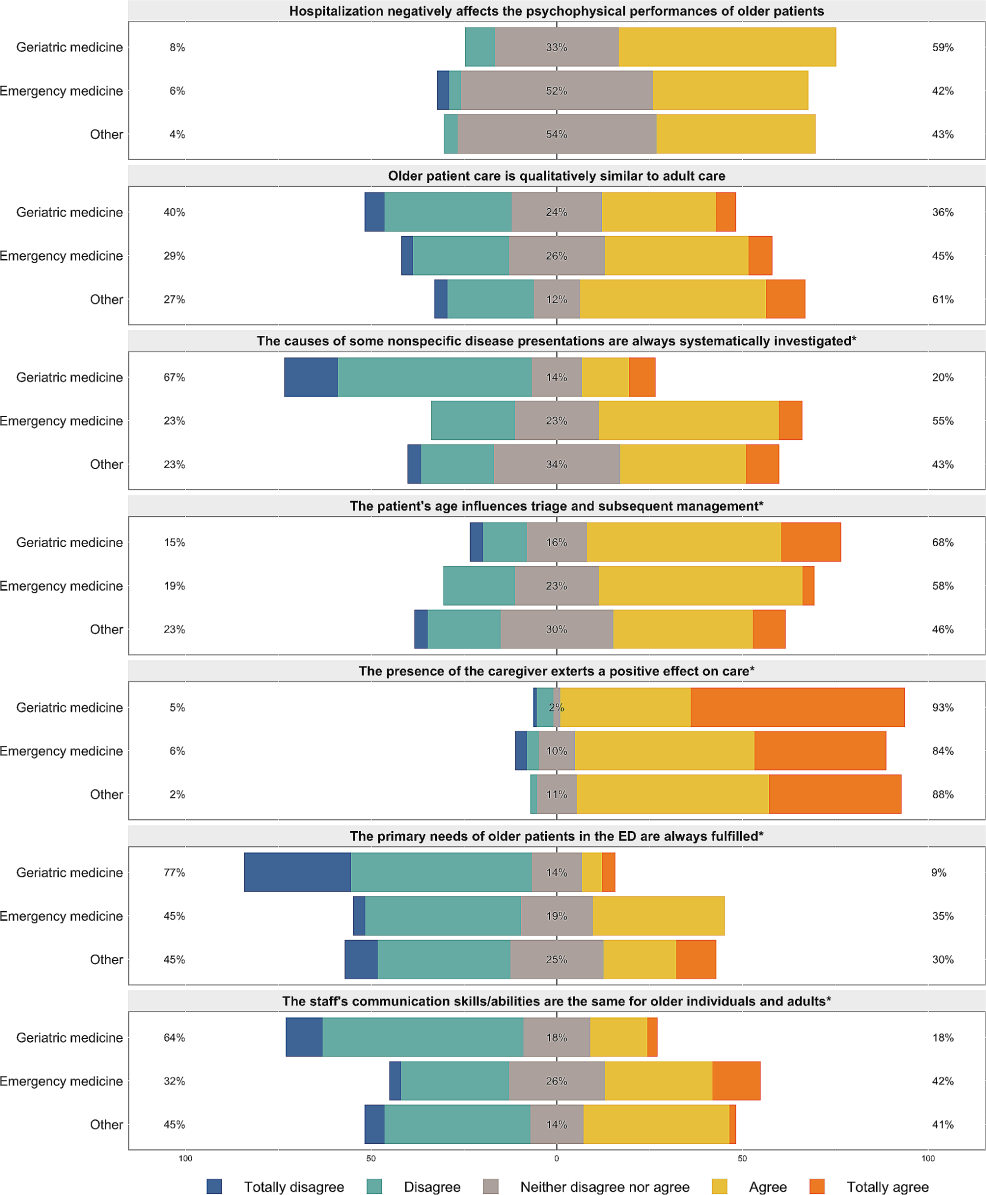
Distribution of older patient care perceptions among Geriatric medicine, Emergency medicine and other specialists on Likert-scale statements. Note: responses were provided on a 5-point Likert scale ranging from 1 *(totally disagree)* to 5 *(totally agree).* *p-value < 0.05

Significant disparities in perceptions of older individuals’ care were observed among different specialists (Fig. 2). Geriatricians exhibited more frequent disagreement regarding the adequate addressing of primary needs in ED compared to others (*p* < 0.001). They also perceived triage and patient care as influenced by age to a greater extent and were more often in agreement with the beliefs that hospitalization has a negative impact on the psychophysical conditions of older people (*p* = 0.029) and that the caregiver’s presence has a positive effect on care (*p* = 0.029). Furthermore, disparities emerged in recognizing the causes of non-specific disease presentations (*p* < 0.001) and in equally evaluating staff skills for older adults and younger patients (*p* = 0.002): in both cases, geriatricians’ opinion was less favorable. Nevertheless, almost all the participants (96.8%) stated that assessing older people at the ED requires more time than evaluating younger patients.

Finally, participants endorsed the implementation of geriatric training for the optimal management of older patients in EDs (97%). This training should be mandatorily included in the emergency medicine residency program according to 98% of geriatricians, as well as 68% of emergency medicine specialists and 73% of other specialists. Most of the remaining respondents suggested it should be an optional pursuit for those interested.

## Discussion

Findings from this study aim to offer a comprehensive view on the current state of care provided to older individuals in Lombardy’s EDs. Despite the availability of several tools to assess the multiple needs of older people attending the EDs, the geriatric risk profile is frequently left unassessed, and comorbidities continue to be the main factor influencing the decision for hospitalization, along with the severity of acute illness. The lack of specific training and dedicated pathways for managing older people accessing the ED is perceived as a cross-cut limitation across various specialties.

Previous literature has shown that the current disease-oriented and episodic model of emergency care inadequately addresses the complex and intricate needs of older people, proving to be unsuccessful. In this context, failure to recognize geriatric syndromes, which manifest as the presenting phenotype of many acute illnesses in older individuals, is associated with patients being assigned to a low-priority category in the ED [30], leading to further adverse outcomes [3, 4, 22, 26].

Our data support the perceived difficulties faced within emergency care for older patients, as demonstrated by the disparities between geriatricians and other specialists in understanding and evaluating symptoms, healthcare needs and hospitalization-related burden. Moreover, they align with the well-documented challenge that delirium often poses in emergency care settings. Despite being present in up to 20% of older adults presenting to the ED, this condition indeed goes frequently unrecognized and overlooked, particularly by non-geriatricians [31, 32].

Patient-centered care has emerged as a priority across diverse healthcare settings [33, 34]. Recently, a systematic review and meta-ethnographic analysis encompassing thirteen articles underscored its substantial value also within the ED context, advocating for a standardization into clinical practice [35]. This transition, contrary to the paradigm of rapid evaluation and referral, reflects a growing recognition of the need to evolve toward a geriatric approach. Various innovative models of care for older adults in ED settings have been developed, all emphasizing comprehensive assessment and tailored care as key points [36, 37]. The Geriatric Emergency Department (GED) stands out as one such model, aiming to recognize patients who may benefit from hospitalization and implement outpatient care for those who do not require it [17, 38]. GEDs, with specialized staff and enhanced environmental features such as better lighting and signage, create a comfortable setting, assisting older adults with mobility problems and reducing delirium risk [39]. Another model is represented by the Frailty Unit, a geriatrician-led multidisciplinary team that supports ED physicians in evaluating frail older patients and enhancing their care pathway [40, 41]. Studies have demonstrated that implementing such models at the ED may improve both patient outcomes and systems-level metrics, by reducing ED length of stay, hospital admission, hospital length of stay and costs, without increasing mortality or readmission [42–44].

In the context of frailty and vulnerability, existing screening tools may be used to identify patients at high risk of adverse outcomes. In particular, the Identification of Seniors at Risk tool (ISAR) and Triage Risk Screening Tool (TRST) have shown comparable results in predicting hospital admission, mortality and early ED revisit in older people presenting to emergency service [45]. In another study conducted on individuals aged 75 years and above attending an Italian geriatric ED, the Silver Code, a tool based solely on administrative data, was demonstrated to offer a prognostic stratification comparable with that obtained with direct patient evaluation [46]. According to our data, no screening tool for identification of high-risk older patients is used in 41.6% of the cases, in line with previous literature [24]. In a recent qualitative evidence synthesis on screening in ED, findings illustrated that staff experiences screening in the ED as a challenging process and that competing interests, environmental stressors such as overcrowding and an organizational culture that resists screening were barriers [47]. Accumulating evidence indicates that implementing screening procedures in the ED enhances the effectiveness of geriatric interventions, helps in allocation of beds for hospitalized patients and directs the most vulnerable individuals toward geriatric evaluation and management units [13, 48]. Finally, screening may raise the awareness of both health care workers and caregivers toward the needs of frail older individuals.

In addition to medical requirements, older patients often present with concurrent functional, psychological and social needs. According to our results, the most frequent reason to consult a geriatrician in the ED was indeed the presence of cognitive-behavioral issues. Given its traditional focus on the rapid evaluation and stabilization of acute conditions, care in emergency settings may not align well with the complexity of individuals affected by cognitive impairment [49]. Previous studies on persons living with dementia indeed demonstrated that they are more likely to receive antipsychotic treatment in the ED and to be hospitalized than older adults without dementia [50]. Moreover, it has been noted that older patients with behavioral disorders disproportionately use the ED [51] and that seniors with cognitive impairment who had a long stay at the ED exhibited more behavioral disorders than those with a short stay [52]. A comprehensive understanding of a patient’s distress is mandatory in this regard, emphasizing the benefits of a geriatric consultation.

In a previous survey conducted among Belgian EDs [24], hospitalization requests were reported as the most frequent reason for geriatric consultation, aligning with our study where it indeed ranked second in frequency.

It has been previously reported that 51% of patients discharged from EDs are unable to complete basic activities of daily living [53]. Functional decline itself appears as an important predictor of further functional decline, hospitalization, length of stay, repeated ED visits, need for home care, institutionalization, and death, surpassing the reason for the patient’s presentation to the ED [54]. Notwithstanding these statistics, functional impairment emerged as one of the least frequently cited reasons for geriatric consultation in our study, potentially indicating a gap in acknowledging and managing this dimension of older patients’ health within emergency care settings. In a cross-sectional study involving 101 randomly selected frail older patients attending four EDs, functional data appeared to be significantly under-documented, lacking in approximately 75% of the cases; additionally, when the information was reported in medical records, it was often not structured or organized [55].

Similarly, literature suggests that processes of transition from ED to home are frequently overlooked, despite growing evidence that older people discharged home from the ED suffer a high rate of adverse health outcomes, as well as increased rate of health service use [3, 12]. In our study, the paucity of pathways aimed at avoiding care fragmentation, as evidenced by the limited involvement of specialized nursing staff in the discharge process and the infrequent direct discharge to nursing homes, emerged as an important barrier to optimal patient management.

The challenges and difficulties encountered in addressing the above-mentioned issues may originate from the widespread need for education in geriatric emergency medicine among participants. EDs represent a unique environment where a deeper comprehension and specialized training in managing the complex aspects of geriatric emergency care play a crucial role in enhancing outcomes for older patients [38]. Literature has consistently reported insufficient training and a general discomfort in clinical decision making when dealing with older patients compared to their younger counterparts presenting with similar complaints [56]. Hesselink et al. studied the influence of a geriatric education program on emergency physicians’ knowledge and attitudes via pre-post tests and patient record analysis, finding beneficial effects for physicians from this teaching [57].

In our study, over 95% of physicians advocated for integrating a geriatric curriculum into standard training, thus underscoring the urgency of developing programs to bridge this educational gap, better preparing healthcare professionals to address complexities presented by this patient population.

This is the first work to investigate current practices and challenges on the management of older patients attending the EDs within the Lombardy region, drawing upon a population and emergency care profile that holds comparable significance to certain European nations. The survey also investigated multiple aspects of this management, focusing on the integration of geriatric assessments, dedicated protocols within and beyond emergency care, and specialized training.

Nevertheless, some potential biases must be acknowledged. Firstly, there may be a sampling bias, as physicians with a greater interest in geriatric issues might have been more likely to participate, particularly among those practicing in EDs. Additionally, the reliance on self-reported data introduces the possibility of influence from factors not considered in the questionnaire, such as individual experiences and contextual circumstances. Secondly, no measures to prevent double entries were adopted. This could potentially impact the accuracy of our findings. Moreover, the survey design employed in this study was pragmatically developed, given the absence of previously validated questionnaires. Nevertheless, meticulous efforts were made to enhance the survey’s validity and reliability through rigorous field-testing procedures. Lastly, although Lombardy represents the most populous Italian region, the unique characteristics and healthcare landscapes of this area may affect the generalizability of our results. In this context, our study may serve as a compelling call to action, urging a comprehensive survey that encompasses various European regions. The replication of our study’s findings would significantly enhance the robustness of the insights gained, not only facilitating the validation of our data but also enabling cross-context comparisons. Such comparisons are instrumental in advancing our understanding of awareness, knowledge, and competencies in geriatric emergency care on a pan-European scale.

## Conclusions

In conclusion, older people represent a substantial portion of ED users and present unique care needs. Findings from this study underscore the imperative for tailored care. Addressing disparities in tool familiarity, perceptions, and discharge processes could significantly enhance the quality of assistance for this vulnerable population, while reducing the strain on EDs. Moreover, the provision of specialized training opportunities is mandatory to ensure comprehensive and standardized management across emergency settings.

### Electronic supplementary material

Below is the link to the electronic supplementary material.

Supplementary Material 1

Supplementary Material 2

## Data Availability

Data are available in anonymous form upon reasonable request addressed to the corresponding author.

## Abbreviations

ED: Emergency Department
GED: Geriatric Emergency Department
ISAR: Identification of Seniors at Risk
TRST: Triage Risk Screening Tool
