# Supplementary material for: Optimizing older patient care in emergency departments: a comprehensive survey of current practices and challenges in Northern Italy
Source: BMC Emerg Med. 2024 May 20;24:86. doi: 10.1186/s12873-024-01004-y (PMC11103964; doi:10.1186/s12873-024-01004-y)
Supplement: Supplementary file 1 — Supplementary Material 1 [file 12873_2024_1004_MOESM1_ESM.docx]

**Additional file 1.** Survey content

1. Gender:
2. Male
3. Female
4. How old are you? _____
5. What is your professional title?
6. Specialist physician (please specify the specialty field)
7. Resident physician (please specify the specialty field)
8. Physician without residency
9. Other
10. In which region do you work?
11. Abruzzo
12. Basilicata
13. Calabria
14. Campania
15. Emilia-Romagna
16. Friuli-Venezia-Giulia
17. Lazio
18. Liguria
19. Lombardia
20. Marche
21. Molise
22. Piemonte
23. Puglia
24. Sardegna
25. Sicilia
26. Toscana
27. Trentino-Alto Adige
28. Umbria
29. Valle d’Aosta
30. Veneto
31. How many years have you been working?
32. Less than 5 years
33. From 5 years to 9 years
34. From 10 years to 20 years
35. More than 20 years
36. What is your predominant job setting?
37. Acute hospital ward
38. Emergency Department
39. Nursing home
40. Rehabilitation facility
41. Intermediate care
42. Outpatient clinic
43. Primary care
44. Locum services
45. Hospice/Palliative care
46. Other (please specify)
47. Do you currently work in a University-related/affiliated hospital?
48. Yes
49. No
50. I don’t know
51. How many beds does your hospital have?
52. Less than 500
53. Between 500 and 1000
54. More than 1000
55. Do you currently work in a hospital that has an Emergency Department?
56. Yes
57. No
58. In the hospital where you work or the one you reference, are there specialized care pathways for managing elderly patients in the Emergency Department?
59. Yes
60. Currently no, but planning is underway
61. No
62. I don't know
63. These care pathways:
64. Follow a formal written procedure
65. Are part of informal/unwritten procedures
66. I don’t know
67. Do you think it's necessary to establish a specific pathway for treating individuals accessing the Emergency Department and residing in nursing homes?
68. Yes, to be organized locally in individual cities
69. Yes, to be organized at a regional level
70. No
71. I don't know
72. Is a consultant geriatrician available in your hospital?
73. Yes, 24h/7
74. Yes, during the daytime
75. Yes, only on certain days of the week
76. No
77. I don't know
78. How often is the consultant geriatrician requested in the Emergency Department?
79. Daily
80. At least twice a week
81. Approximately once a week
82. Less than once a week
83. Never
84. I don't know
85. How often is the consultant geriatrician requested in the Emergency Department for each of the following reasons?

|  | Never | Rarely | Sometimes | Often | Always |
| --- | --- | --- | --- | --- | --- |
| 1. Unclear need for hospitalization |  |  |  |  |  |
| 1. Admission to geriatric ward |  |  |  |  |  |
| 1. Motor deficits |  |  |  |  |  |
| 1. Functional impairment |  |  |  |  |  |
| 1. Cognitive-behavioral issue (e.g., dementia, agitation, stupor, confusion) |  |  |  |  |  |
| 1. Need for social context assessment |  |  |  |  |  |

1. What screening tool **do you know** to identify patients with a geriatric risk profile?
2. Triage Risk Screening Tool (TRST)
3. Identification of Seniors at Risk (ISAR)
4. InterRAI
5. Silver Code
6. Other (please specify)
7. None
8. What screening tool **is used** to identify patients with a geriatric risk profile in the Emergency Department of the hospital where you work or the one you reference?
9. Triage Risk Screening Tool (TRST)
10. Identification of Seniors at Risk (ISAR)
11. Inter-RAI
12. Silver Code
13. Other (please specify)
14. None
15. I don't know
16. Who most often fills in the tool to identify patients with a geriatric risk profile in the Emergency Department of your hospital?
17. Emergency department nurse
18. Emergency department doctor
19. Geriatrician
20. Automated compilation
21. Other (please specify)
22. I don't know
23. Besides the severity of acute illness, how important do you consider the following parameters in determining the hospitalization of an elderly patient?

|  | Very unimportant | Unimportant | Neutral | Important | Very important |
| --- | --- | --- | --- | --- | --- |
| 1. Comorbidities |  |  |  |  |  |
| 1. Moderate to severe cognitive deficits |  |  |  |  |  |
| 1. Behavioral disturbances |  |  |  |  |  |
| 1. Delirium |  |  |  |  |  |
| 1. Motor deficits |  |  |  |  |  |
| 1. Bedridden for at least 3 months |  |  |  |  |  |
| 1. Recent fall |  |  |  |  |  |
| 1. Repeated hospital admissions |  |  |  |  |  |
| 1. Coming from a nursing home |  |  |  |  |  |
| 1. Under palliative care service |  |  |  |  |  |
| 1. Difficulties of territorial medicine |  |  |  |  |  |
| 1. Poor social support |  |  |  |  |  |

1. In the discharge process of an elderly patient from the Emergency Department of the hospital where you work or the one you reference, are involved:
2. Nurses specialized in assisted discharge
3. Generalist nurses
4. Both nurses
5. None of the nurses
6. I don't know
7. In the hospital where you work or the one you reference, is the procedure of directly transferring a patient to a nursing home utilized?
8. It is an established practice
9. It has rarely occurred, in selected cases
10. Never
11. I don't know
12. To what extent do you agree with the following statements?

|  | Totally disagree | Disagree | Neither disagree nor agree | Agree | Totally agree |
| --- | --- | --- | --- | --- | --- |
| 1. In the Emergency Department of your hospital, elderly care is qualitatively similar to adult care |  |  |  |  |  |
| 1. Hospitalization negatively impacts the psychophysical performances of elderly patients |  |  |  |  |  |
| 1. The causes of some nonspecific disease presentations (e.g., delirium and falls) are always systematically investigated |  |  |  |  |  |
| 1. The patient's age influences triage and subsequent care. |  |  |  |  |  |
| 1. The presence of a caregiver inside the Emergency Department and their proactive participation, especially for patients with cognitive deficits, has a positive effect on care |  |  |  |  |  |
| 1. The causes of some nonspecific disease presentations (e.g., delirium and falls) are always systematically investigated. |  |  |  |  |  |
| 1. During the stay in the Emergency Department, the primary needs of elderly patients (meals, hygiene, urination, and defecation) are always fulfilled |  |  |  |  |  |
| 1. The staff’s communication abilities are the same for elderly and adults |  |  |  |  |  |

1. Do you believe that assessing an elderly patient in the Emergency Department requires more time than assessing a young patient?
2. Yes
3. No
4. I don't know
5. Do you think a specific training program is necessary for managing elderly patients in the Emergency Department?
6. Yes, to be conducted as part of the Emergency Medicine residency program
7. Yes, optional for those interested
8. No
9. I don’t know
