# Supplementary material for: Optimizing older patient care in emergency departments: a comprehensive survey of current practices and challenges in Northern Italy
Source: BMC Emerg Med. 2024 May 20;24:86. doi: 10.1186/s12873-024-01004-y (PMC11103964; doi:10.1186/s12873-024-01004-y)
Supplement: Supplementary file 2 — Supplementary Material 2 [file 12873_2024_1004_MOESM2_ESM.docx]

**Additional file 2.** Differences in factors determining older patients’ hospitalization among Geriatric medicine, Emergency medicine and other specialists


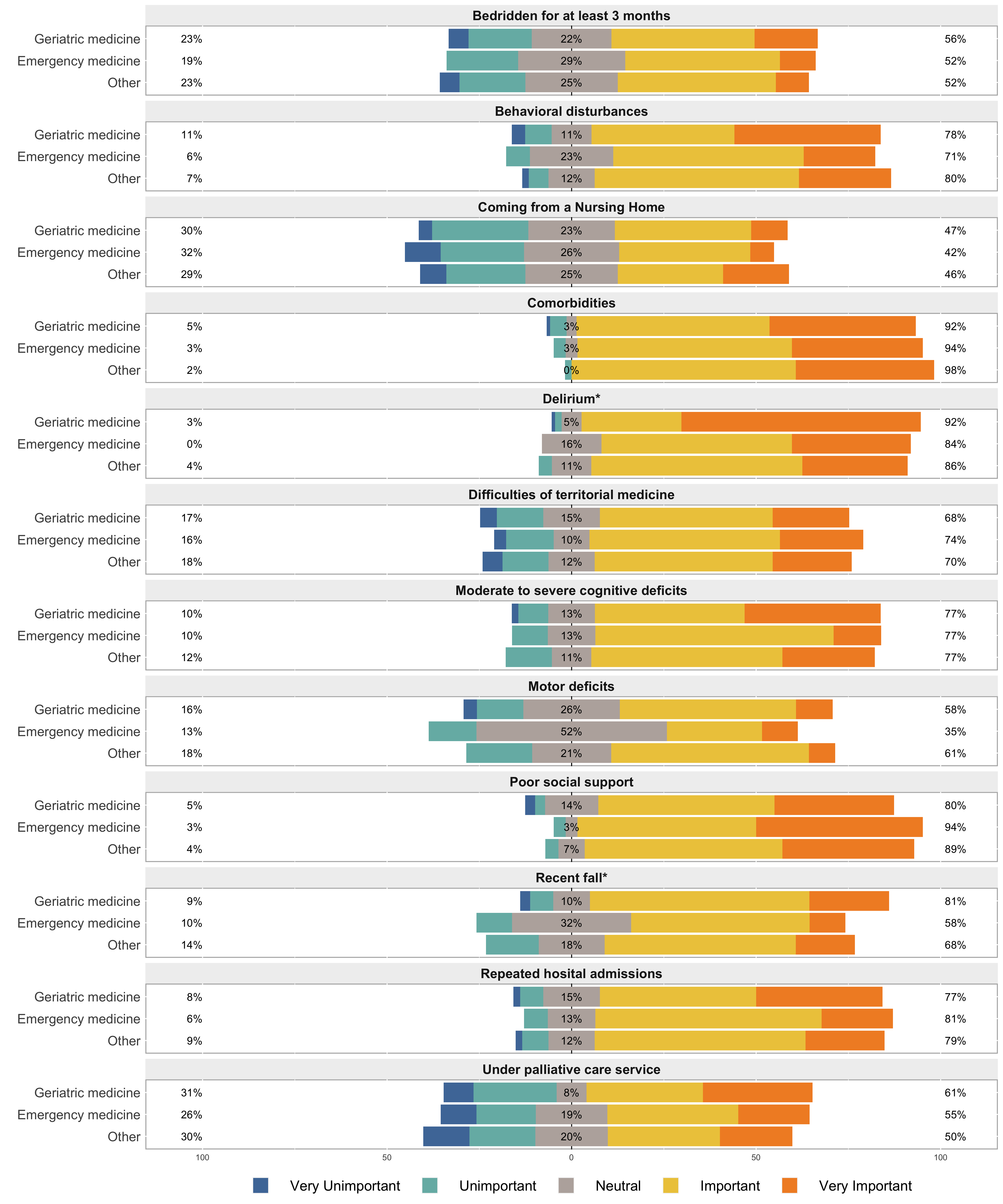


Note: *p-value < 0.05
